# Partially Unroofed Coronary Sinus, Persistent Left Superior Vena Cava and Cortriatriatum: A Rare Combination of Interruption in Normal Embryogenesis

**DOI:** 10.5812/cardiovascmed.15383

**Published:** 2014-02-24

**Authors:** Mohammadmehdi Peighambari, Maryam Esmaeilzadeh, Azin Alizadehasl, Nehzat Akiash, Mahmoodreza Motamedzadeh

**Affiliations:** 1 Cardiovascular Intervention Research Center, Rajaie Cardiovascular Medical and Research Center, Iran University of Medical Sciences, Tehran, IR Iran; 2 Echocardiography Research Center, Rajaie Cardiovascular Medical and Research Center, Iran University of Medical Sciences, Tehran, IR Iran; 3 Cardiovascular Imaging, Ultrasound Research Lab, Tufts Medical Center, Boston, USA

**Keywords:** Coronary Sinus, Cor Triatriatum, Echocardiography

## Abstract

A 48-year-old male with a history of secundum type atrial septal defect (ASD) closure in childhood presented to our outpatient clinic complaining of palpitation for six months. Interestingly, transthoracic and transesophageal echocardiography exams showed an undiagnosed partially unroofed coronary sinus associated with persistent left superior vena cava (LSVC) and Cor triatriatum.

## 1. Introduction

Unroofed coronary sinus (CS) is a rare congenital cardiac defect defined as partial or complete absence of the partition separating the coronary sinus and the left atrium. Unroofed CS is often associated with other congenital abnormalities. Patients may present with dyspnea, positional or transient cyanosis, hypoxia, paradoxical embolus, cerebral abscess or right-sided heart enlargement and failure or they may be completely asymptomatic (1, 2). The case presented here is a 48-year-old patient with a history of surgical closure of an atrial septal defect (ASD) in whom an unroofed coronary sinus was discovered during work up for palpitation.

## 2. Case Presentation

**Figure 1.**
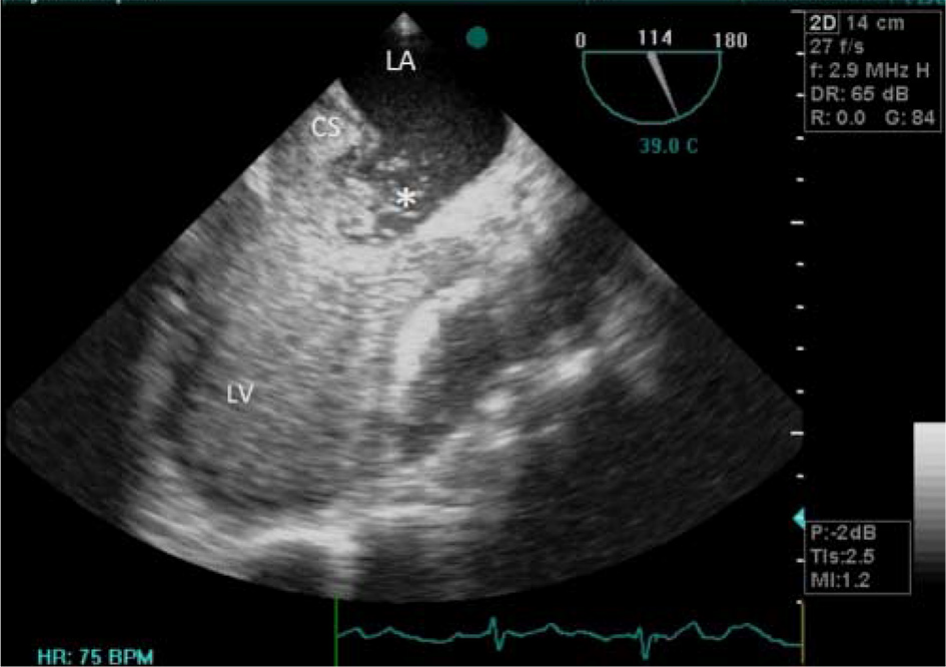
Transesophageal Echocardiography at 115 Degree Showing Large Defect (Asterisks) in Mid Part of Coronary Sinus With Significant Left to Right Shunt (Negative Contrast) During Agitated Saline Injection CS, coronary sinus; LA, left atrium; LV, left ventricle.

**Figure 2.**
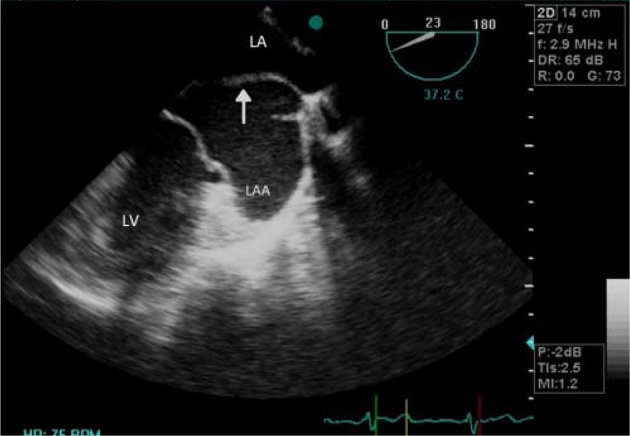
Transesophageal Echocardiography Showing Membrane Within Left Atrium (Arrow), Just Above Left Atrial Appendage LA, left atrium; LAA, left atrial appendage; LV, left ventricle.

A 48-year-old male presented to our outpatient clinic complaining of six months history of palpitation. He reported no previous history of chest pain, dyspnea, cyanosis, or syncope. His past medical history included surgical ASD (secundum type) closure performed at the age of 12. He had no coronary artery risk factors. On physical examination there was not cyanosis or clubbing. On cardiovascular examination, the heart rhythm was atrial fibrillation, without abnormal finding in auscultation. A routine transthoracic echocardiography (TTE) was done that revealed normal left ventricular size with preserved systolic function (LVEF = 55%). Right ventricle was mildly enlarged with mild systolic dysfunction. Mild to moderate tricuspid regurgitation was also detected along with mild pulmonary artery hypertension (systolic pulmonary artery pressure = 35 mmHg) and mild pulmonary insufficiency. Agitated saline solution injection in to the left arm demonstrated substantial early bubble passage into the left atrium (LA). Hence, transesophageal echocardiography (TEE) was performed that showed a large defect (2.0 cm) in the mid part of coronary sinus (Figure 1) with significant left-to-right shunt by color flow Doppler (QP/QS = 1.5). Persistent left superior vena cava (LSVC) draining into the coronary sinus was also identified. Residual shunt from surgically closed secundum ASD was not evident by color flow Doppler A nonrestrictive membrane was seen within LA without any turbulent flow or gradient just above left atrial appendage (LAA); the LAA was aneurysmally dilated (Figure 2) due to the flow from pulmonary veins toward it. The patient underwent cardiac catheterization for shunt quantification and then referred for surgical correction. Coronary sinus defect was closed surgically with pericardial patch and patient discharged uneventfully after a week.

## 3. Conclusions

An unroofed CS is a rare cardiac abnormality in which the CS communicates directly with the left atrium (LA) because of a congenital defect in the tissue separating the LA and the CS. This lesion was considered as the rarest type of atrial septal defect (1). Unroofed CS is often seen associated with other congenital heart disease, particularly persistent left superior vena cava (LSVC) that drains directly into the coronary sinus (1, 2); however, other types of congenital heart disease such as Cor triatriatum, pulmonary atresia, tetralogy of Fallot, and anomalous pulmonary venous drainage are reported in patients with coronary sinus defects (3). It is most commonly diagnosed in childhood. Although the most common associated anomaly with unroofed coronary sinus is persistent left superior vena cava (which drains into the right atrium through the orifice of the coronary sinus), isolated unroofed coronary sinus can occur (4). Four different morphologies of unroofed CS have been defined (1). In type I, completely unroofed CS is associated with persistent LSVC. Type II is a completely unroofed CS without persistent LSVC. In type III, CS is partially unroofed in its mid portion and in type IV, absence of the terminal portion of the CS creates a partially unroofed CS. Unroofed CS typically communicates with the LA at the junction of the LA appendage and the left upper pulmonary vein. The clinical presentation is largely determined by the size of the defect and the degree of left-to-right shunting. Patients may be completely asymptomatic or present with symptoms ranging from nonspecific complaints to symptoms of right-sided heart failure due to chronic right ventricular volume overload. Transthoracic echocardiography is typically used as the first imaging modality for suspected unroofed CS.

Generally, lacks of the ability to visualize the posterior cardiac structures, such as the CS and pulmonary veins, is a major disadvantage of TTE. Accordingly, TEE that is able to assess these posterior structures more accurately, particularly in mid-esophageal long-axis views, often confirms the anomaly (5). Recently, multidetector computed tomography (MDCT) and cardiac magnetic resonance imaging have provided promising results (2, 6-8). In addition, several arrhythmias have been also reported in patients with unroofed CS. Wolff Parkinson White (WPW) syndrome was diagnosed in a 64-year-old man with unroofed CS and 30-year history of palpitations (9). Atrial tachycardia has also been found in a two-year-old girl with Cor triatriatum, unroofed coronary sinus, and persistent left superior vena cava (10). However, atrial fibrillation associated with an unroofed CS and a persistent LSVC has not been reported yet. Cardiac anatomy should be carefully evaluated in patients with history of congenital/anatomical heart disease, since unroofed CS may be detected singly or in association with other congenital heart defects. It may be even incidentally detected during cardiac evaluation; therefore, being aware about this anomaly has a paramount importance for proper diagnosis.
